# Use of Complementary and Alternative Medicine for Epileptic Children in Tehran: A Cross-Sectional Study (2009-2011)

**Published:** 2014

**Authors:** Seyed Hassan TONEKABONI, Sepideh JAFARI NAEINI, Ali KHAJEH, Omid YAGHINI, Ahad GHAZAVI, Fatemeh ABDOLLAH GORJI

**Affiliations:** 1 Pediatric Neurology Research Center, Shahid Beheshti University of Medical Sciences (SBMU), Tehran, Iran; 2 Pediatric Neurology Excellence Center, Pediatric Neurology Department, Mofid Children Hospital, Faculty of Medicine, Shahid Beheshti University of Medical Sciences (SBMU), Tehran, Iran; 3 Clinical Research Development Center, Mofid Children’s Hospital, Shahid Beheshti University of Medical Sciences (SBMU), Tehran, Iran

**Keywords:** Epilepsy, Complementary and Alternative Medicine (CAM), Children

## Abstract

**Objective:**

Although the use of Complementary and Alternative Medicine (CAM) has been evaluated globally, there are few studies in our country on this subject. The purpose of this study was to determine the prevalence, pattern of use, parental sources of information, and benefits of CAM in epileptic children in Tehran.

**Materials & Methods:**

One hundred thirty-three parents or relatives of epileptic children who were referred to outpatient clinics or admitted in neurologic ward of four major hospitals in Tehran, were interviewed by our researcher based on a structured questionnaire; from 2009 to 2010. The information obtained comprised the demographic data of patients and their parents, frequency and morphology of convulsions, the type and sources of CAM and finally, the benefits and adverse effects of this practice.

**Results:**

Forty-four percent of the respondents had used CAM methods either alone or in combination with other methods. The most frequently used CAM was written prayers followed by oral herbs and special diets. CAM was mainly introduced to them by relatives. Only 16.7% of these parents had discussed this matter with their children’s physicians. No efficacy to control seizure was observed for most of these methods.

**Conclusion:**

This study showed that use of CAM in our study group is relatively common and may have a potentially hazardous role in the treatment process. So, it is necessary for physicians to have enough information about CAM practice in their patients.

## Introduction

Complementary and Alternative Medicine (CAM), according to the definition of American National Institute of Health (NIH) comprises medical treatments that are not included as traditional medicine (1), although there have been other definitions too. In World Health Organization (WHO) statement in 2003 (2), “Complementary, alternative, unusual and traditional care are types of uncommon management that have been used in different situations with variable efficacy”. 

Use of CAM has been increasing in different countries, and a prevalence of 80% has been reported in some developing countries (3).

The national center for Complementary and Alternative Medicine (NCCAM), in a study in 2004 reported that 36% of adults in the United States used CAM (4). In a study in 2008, this figure has been reported as 56% (5). In patients with chronic disorders and in a majority of patients with neurologic and psychiatric disorders, the tendency towards using CAM is higher (6,7).

Epilepsy as a chronic disease has financial and emotional burdens for family of the affected child. This disease as a result of influencing different aspects of the patient’s life causes problems and limitations. Based on literature, 30-40% of the epileptic patients suffer from refractory epilepsy (8), in which propensity towards CAM is higher due to underlying brain damage and chronic antiepileptic drugs (AEDs) consumption. 

In our country, there have been no accurate statistical reports concerning the use of CAM and its impact

on treatment in children and adults suffering from epilepsy. A prevalence of 38% in epileptic children (9) and 39% in adults have been reported in a limited study (10).

Recently, there has been a rise in the use of this type of treatment and high prices have been paid for it in Tehran (11). The increase in tendency towards this branch of medicine, unknown probable complications, and also parents’ avoidance to consult their physicians before using CAM, may interfere with the treatment process. The objective of this study was to evaluate the frequency and types of different CAM therapies in children with seizure in four major hospitals in Tehran. Meanwhile, we assessed the potential benefits and complications of CAM therapy.

## Material & Methods

A total of 133 patients with seizure disorder aged 1 month to 16 years, who were visited and followedup at neurology clinics or admitted to the neurology wards of four hospitals in Tehran, were included in this descriptive study from 2009 to 2011. Three of these hospitals (Mofid, Aliasghar, and Children Medical Center) are major children’s hospitals and Imam Hossein hospital is a general hospital situated in a highly populated district. A questionnaire consisted of demographic information of the patients and their parents, frequency of convulsions, types of CAM, as well as sources, benefits, and adverse effects of this practice. in addition, they were asked how CAM was introduced to them and whether they had consulted their physicians about it. The reliability and validity of the questionnaire was confirmed by a primary test on 15 case samples and subsequently asking the neurologists’ point of view. All parents were completely informed of their children’s treatment process and a minimum of 1 month had passed from the beginning of their disease. Seizure disorder was defined as at least two recurrent unprovoked seizures originating from paroxysmal discharges of cerebral neurons. By this definition, occasional seizures, including febrile, CNS infections, and hypoglycemia were excluded from the study. 

Statistical analysis was performed using IBM SPSS (version 20). All variables of the patients were first summarized by descriptive statistics. Associations of using CAM and the parent’s education level and employment status and the level of familiarity with CAM were analyzed using Pearson chi-square test.Significance level was determined at p<0.05.

## Results

One hundred thirty-three questionnaires were filled out. Ninety-two patients (69.2%) were visited as outpatients and the remaining were hospitalized in the pediatric neurology ward. The mean age of the patients was 76.6 52 months (range, 1 month to 16 years) and 70 (53%) cases were girl.

Ninety-seven percent of the responders were the children’s parents and the remaining 3% were the elder siblings. 85.6% of the patients lived in the urban and 14.4% were residents of the rural area. Table 1 demonstrates the parents’ education and employment status.

**Table 1:** Parents’ Education and Employment Status

	Number	Percent
Education (n=130)		
No education	2	1.5%
Primary or secondary school	108	83%
University or postgraduate degree	20	15.5%
Employment status (n=131)		
Yes	129	98.5%
No	2	1.5%

The mean age at disease onset was 34.6 40.7 months (range, 0-156 month) and the mean duration of the disease was 42.7 41.1 months (range, 1-174 months). The most frequent seizure type was generalized toniccolonic seizure (primary or secondary) observed in 110 patients (90.2%) followed by complex partial, simple partial, or mixed type seizures, (7.4%, 1.6%, and 0.8%, respectively).

The mean number of seizure attacks per month at the time of visit was 37.4 86.2. Monotherapy was used in 62 (47.3%) patients, and 33 (25.2%) cases used more than two AEDs. The mean number of prescribed AEDs for each patient was 1.9 1.0.

Of the 133 respondents, 59 (44.4%) families used CAM for their children. 18.2% of the patients continued CAM for treatment of their seizures during the last month of filling out the questionnaire. Twenty-four (40.7%) families in the group of CAM users had tried CAM only for their child’s seizure and the remaining had used CAM for other diseases too. 

Seventy-four cases had never used CAM, while 27 (36.5%) families had considered using this type of treatment, but had never practiced it. Most of the physicians (83.3%) did not know that their patients were under CAM treatment; 34% of parents thought that it was not really necessary to do so; 23.4% believed that CAM practice was a personal concept not related to physician responsibilities; 17% were worried about the negative reaction of the doctors; and the remainder had a combination of these justifications. In the group of parents who consulted their physician about CAM practice, 33.4% were confronted with a dissident response.

The majority of parents (higher than 90%) were aware of only less than 50% of the methods mentioned in the questionnaire. Regarding the source of information about CAM, of the 120 cases analyzed, 102 (85%) parents mentioned that they were influenced by relatives, friends, or neighbors. Media (television, radio, and newspaper) (5.8%), physician and health staff (2.5%) and previous personal experience (6.7%) were the sources of information about CAM practice in the remainder.

Most of the participants (56%) had no information about the cost of CAM and 55% of those who knew, indicated that the cost of CAM treatment was less than that of AEDs.

Forty-one parents mentioned recommendation by relatives as the main reason they used CAM treatment. Many of the parents had more than one answer to this question, because in some families two or more reasons for CAM practice coexisted. The other reasons are demonstrated in Table 2.

**Table 2:** Different Reasons for CAM Practice in 59

Reasons of CAM use	Number
**Recommendation by relative**	41
**No response to AEDs**	25
**Successful experience of family and relative**	19
**Advertisement**	2
**Medication side-effects **	1
**Others (family ritual and …)**	12

66.5 percent of CAM users believed that CAM treatment had no benefit in the management of epilepsy. 27.3% of all participants believed that CAM had no risk. Most of the parents (66.4%) had no propensity towards replacement of AEDs by CAM. Most of the parents (84%) who used CAM, started this practice after using antiepileptic medication, some of whom reported an unfavorable response. Twenty-seven parents (33.7%) supposed that their children’s seizures became under complete or partial control after CAM practice. 

Forty-three (72.9%) patients used only one type of CAM, while 16 (27.1%) cases used two or more types of CAM. The mean number of CAM methods used for each patient was 1.4±1.0.

Among the 59 children who used CAM, a total of 80 CAM methods were tried (Table 3). written Prayer and wearing amulets were the most frequent types of CAM (42 and 52.5%, respectively) and dietary therapy and herbal remedies were used in 12 children (15%), separately. No side-effect for CAM was reported by parents.

There were significant associations between the father’s level of education and familiarity with the CAM therapy (p<0.001) and consideration of using CAM practice (p<0.001). In addition, there were significant associations between the level of familiarity with the CAM method and the employment status of mothers (p=0.008) and fathers (p<0.001) of the patients.

**Table3:** Frequency, Types, and Helpfulness of CAM Therapy Practiced By Parents

**Types of CAM**	**Helpful**	**Not helpful**	**Total**
**Dietary therapy**	8 (66.7%)	4 (33.3%)	12 (15%)
**Herbal remedies**	3 (25%)	9 (75%)	12 (15%)
**Vitamin therapies**	3 (75%)	1 (25%)	4 (5%)
**Massage**	0 (0%)	1 (100%)	1 (0.8%)
**Acupuncture**	0 (0%)	1 (100%)	1 (0.8%)
**Homeopathy**	1 (100%)	0 (0%)	1 (0.8%)
**Cupping**	0 (0%)	1 (100%)	1 (0.8%)
**Written prayer & wearing amulets**	11 (26.2%)	31 (73.8%)	42 (52.5%)
**Energy therapy**	0 (0%)	1 (100%)	1 (0.8%)
**Superstitious practice**	1 (20%)	4 (80%)	5 (8.5%)
**Total**	27 (33.75%)	53 (66.25%)	80 (100%)

## Discussion

The results of the presesent study show that parents of epileptic children, similar to parents of children with other medical conditions practice CAM therapies alongside conventional treatment. In our study, 44.4% of the parents used some kind of CAM for their children. In Nigeria, this percentage was 38% (9). In Turkey, 27.7% of the parents used alternative treatment, excluding religious and nonreligious spiritual practice (12).

In our study, we did not consider all religious practices as CAM. Prayer was not included as CAM, and only written prayer, for which parents paid money to a third person was included. In a survey in Canada, 39% of the epileptic children who were referred to a pediatric neurology clinic received CAM (13). In this study, the source of information for using CAM was mostly family and friends (85%), while the role of media and books was only 12.5%. The physician and health workers had the least influence (2.5%). In Turkey, in 55.6% of the cases, the immediate circle of family and friends was the information source and their doctors’ suggestions were responsible in 6.9% of the cases (12). In Nigeria, 79% of the patients were influenced by relatives and friends to use CAM for their children (9). 

The most frequent reason for using CAM in our patients was success stories told by relatives and media (69.5%), followed by dissatisfaction from conventional medical treatment (42%). In a Canadian study (13), an efficient family experience and the media accounted for 46% and dissatisfaction with AEDs for 14% of the patients.

In our study, 33.7% of families believed that CAM therapy was beneficial (i.e., partial or complete control of seizure). In the Canadian study, this belief was observed in 71% of the patients’ caregivers (13) and it was reported in 51.6% of the Turkish parents (12). The usefulness of CAM was only the parents’ belief, and as no family withdrew their children from conventional AED, the seizure control may have been due to AED or the natural clinical course of epilepsy. 

The most frequent CAM method in our patients was “written prayer” and “wearing amulets” (42%), followed by special diet (14%) and herbal traditional remedies (12%). In the study conducted in Turkey (12), religious practices composed 93.8% of CAM, while most of them (89.1%) were regular prayers. But we did not include regular religious practice and prayer as CAM. Other religious practices were used by 40.3% of the parents (12), very similar to our experience, while in the Canadian study, only 12.5% of the caregivers used this method (13).

Special diet and vitamins were practiced in 17.1% of Turkish families (12) and 25% of Canadian caregivers (13). In our study, 14% of the parents used this practice. 

Herbal medicine was used in 12% of our patients, slightly more frequent than the Turkish (7%) (12) and Canadian (7.8%) families (13). 

Some superstitious methods, which were reported in 5% of our patients, such as cracking eggs (3%), putting a gold piece in water and drinking it (1%), and finally passing salt under the child’s clothes, was not reported in other studies, to the best of our knowledge. 

Despite the mentioned reports regarding the sideeffects of CAM (14), no harmful reaction was noticed by parents in our study. This may be due to the limited and conservative use of herbal and traditional remedies by caregivers. 

The samples in our study were limited to four major hospitals in Tehran, and these results cannot be generalized to all Iranian epileptic children, due to the vast ethnic and cultural variety in our country. 

This study showed that use of CAM in our study group was relatively common. 

We suggest that CAM may have a potentially hazardous role in the treatment process. So, it is necessary for physicians to have enough information about CAM practice in their patients.

## Footnote

***Director in Chief ‘s Comments:**

Complementary and Alternative Medicine (CAM) for epileptic children in Tehran, I felt highly necessary to make the following important comments:

1. In Islamic Education, it is believed that” Nothing will happen unless has Almighty God’s order” so it is definite if a medicine cures an illness that cure comes from God’s will.
2. Pray; asking Allah, for something to occur, is one of the most fundamental beliefs of Moslem People. In our Holy book ”Quran” Allah says “You just ask me, I’ll give it to you, if it is in your benefit...” (Soureh Ghafer Verse 60). Also our Prophet (Peace be upon him) said “Prayer is the essence of God’s slavery”
3. The one who prays, should know that his/her prayer will be heard, if he/she is in direct and sincere contact with God. **There is no need to pay someone to write prayer for someone else or use amulet. **
4. Prayer preferably, should use those words which were used by Prophet or Imam, even any one can pray sincerely at any place by any language.
5. We should believe that when we ask God to have something, that request will be materialized, if it is of our own benefit, otherwise God will give us instead many other blessings and benefits either in our life or in the Day of Judgment or both.
6. In Islamic education we are thought that anyone who wants something, should work hard to reach that thing, but in the same time should pray and ask God’s help for implementation of goals if those are safe and beneficial for him/her.

**Mohammad Ghofrani MD**

1.Professor of Pediatric Nerology, Shahid Beheshti University of Medical Sciences, Tehran, Iran

2. Director in Chief of Iran J Child Neurol
